# Production and Peripheral Roles of 5-HTP, a Precursor of Serotonin

**DOI:** 10.4137/ijtr.s1022

**Published:** 2009-03-30

**Authors:** Kazuhiro Nakamura, Hiroyuki Hasegawa

**Affiliations:** 1 Department of Pathology, Juntendo University School of Medicine, Tokyo 113-8421, Japan; 2 Department of Biosciences, Teikyo University of Science and Technology, Uenohara, Yamanashi 409-0193, Japan

**Keywords:** 5-HTP, serotonin, TPH, BH4

## Abstract

Serotonin (5-hydroxytryptamine [5-HT]) has been implicated in a variety of physiological and pathological functions. Multiple steps of enzyme reactions enable biosynthesis of 5-HT. The first and rate-limiting step of the reaction is the synthesis of 5-hydroxy-*L*-tryptophan (5-HTP) from *L*-tryptophan. This step is dictated by an enzyme, tryptophan hydroxylase (TPH). TPH requires 6R-*L-erythro*-5,6,7,8-tetrahydrobiopterin (BH4) as a co-substrate of TPH. 5-HTP has been simply regarded as a precursor of 5-HT and it is believed that the biological significance of 5-HTP is essentially ascribed to the production of 5-HT. However, recent works shed light on the specific functions of 5-HTP in the periphery. In this review article, we focus on the specific roles of exogenous 5-HTP as well as the endogenous 5-HTP in the gut epithelial cells. Since systemic treatment with 5-HTP is applied to patients with lower 5-HT levels, the studies on the specific role of 5-HTP might create an opportunity to explore the effects of exogenously-applied 5-HTP in the gut in man.

## Introduction

Serotonin (5-hydroxytryptamine [5-HT]) has been implicated in a number of physiological and pathological functions in the central nervous system as well as several peripheral organs and tissues such as the liver, platelets and immune systems.^1^–^6^

In the periphery, a majority of 5-HT is produced and stored in the enterochromaffin cells in the gut.^7^ Part of 5-HT is also stored in the platelets that release 5-HT in multiple peripheral organs. It has been believed that the released 5-HT essentially exerts the biological effects via 5-HT receptors on various cells in a paracrine fashion. There are many types of 5-HT receptors that are distributed widely among endocrine, cardiovascular, immune, and gastrointestinal tissues. Receptors for 5-HT fall into one of four distinct families (5-HTR1, 5-HTR2, 5-HTR3, 5-HTR4-7), which are characterized by different signal transduction and physiological roles. Numerous studies have suggested associations between various neuropsychiatric disorders and genes that modulate 5-HT neurotransmission such as the 5-HT transporter and 5-HT receptors.^8^,^9^ Therefore, transport of 5-HT and signal transduction through 5-HT receptors play direct roles in neuronal functions elicited by 5-HT. In fact, selective serotonin reuptake inhibitors are used for the treatment of several psychiatric disorders.^9^,^10^

Administration of 5-hydroxy-*L*-tryptophan (5-HTP), a 5-HT precursor can be used to correct 5-HT concentration in the brain of some patients with phenylketonuria.^11^ Hyperphenylalaninemia seen in phenylketonuria is associated with a decrease in availability of biogenic amines in the brain.^12^ Early studies reported reduced levels of dopamine, norepinephrine and 5-HT in post mortem brain tissue,^13^ and recent reports indicate reduced aminergic synthesis in the brain of adult phenylketonuria patients on phenylalanine-free diet^14^ and in mild hyperphenylalaninemia with neurological signs.^15^ Although 5-HTP has been detected biochemically^16^ and 5-HTP-immunoreactivity has been visualized in neurons,^17^–^19^ it has been generally assumed that 5-HTP is immediately decarboxylated to 5-HT and consequently little 5-HTP exists in the CNS.^20^

Although the specific functions of 5-HTP in the brain have not been reported, some works shed light on specific functions of 5-HTP in the extraneuronal cells.^21^,^22^ This article reviews the mechanisms of 5-HTP synthesis by focusing on 6R-*L-erythro*-5,6,7,8-tetrahydrobiopterin (BH4), a co-substrate for tryptophan hydroxylase (TPH). Then, the specific role of 5-HTP in the periphery that is independent of 5-HT is discussed.

## Production of 5-HTP

In the pathway of 5-HT biosynthesis, TPH, a member of a family of pterin-dependent aromatic amino acid hydroxylases, catalyzes the formation of 5-hydroxy-*L*-tryptophan (5-HTP) from *L*-tryptophan, which is the first step in the biosynthesis of the neurotransmitter 5-HT.^23^–^26^ Aromatic *L*-amino-acid decarboxylase (AADC) subsequently mediates the production of 5-HT. TPH is a mono-oxygenase, which incorporates one atom of oxygen from molecular oxygen into the substrate and reduces the other atom to water. The two electrons required for the reduction of the second atom to water are supplied by BH4. BH4 acts as the co-substrate for TPH rather than as a tightly bound cofactor.^25^,^26^ Since Km of TPH for BH4 is rather large, TPH activity is dependent on cytosolic concentration of the co-substrate BH4. The mechanism of how the cytosolic level of BH4 is maintained is therefore important in the understanding of 5HT production in a living cell.

To date, complete deficiency of TPH activity has not been described in man.^27^ But inherited disorders affecting BH4 metabolism, in general, lead to severe deficiency of 5-HT as well as dopamine within the central nervous system. The exceptions are dominantly inherited GTP cyclohydrolase deficiency, where only dopamine metabolism is affected, and pterin 4a-carbinolamine dehydratase deficiency and milder forms of 6-pyruvoyltetrahydropterin synthase deficiency, where neurotransmitter metabolism appears normal.^27^ Thus, the focus was on the kinetics of BH4 that highly influences TPH activity and 5-HTP production. Factors regulating TPH activity other than BH4, such as phosphorylation and ferrous iron, are essentially introduced in other articles.^28^–^32^

BH4 is also an essential cofactor for aromatic amino acid hydroxylases of phenylalanine^33^ and tyrosine,^34^ fatty acid glycerylether oxygenase,^35^ and nitric oxide (NO) synthase.^36^,^37^ The biosynthetic pathway of BH4 involves at least 4 essential enzymes, GTP-cyclohydrolase I, 6-pyruvoyl-tetrahydropterin synthase, 6-pyruvoyl-tetrahydropterin 2′-reductase and sepiapterin reductase.^38^–^40^ The immediate precursor of BH4 in the *de novo* synthetic pathway is believed to be 6-lactoil-tetrahydropterin, a substrate of sepiapterin reductase which catalyzes reduction of a ketone to hydroxyl of the C6-side chain of either tetrahydro- or dihydro-form of pterin, respectively. Sepiapterin is a dihydro form of 6-lactoil-pterin. Since biopterin-deficient mutants of *sepia* of a fruit fly *Drosophila melanogaster* and *lemon* of a silk worm *Bombyx mori* deposited Sepiapterin, this pterin was long believed to be the intermediate in biopterin biosynthesis.^41^

BH4 supplementation has been used in BH4-deficient patients for a long time.^42^ BH4 supplementation is used in BH4-deficient patients to decrease phenylalanine levels in patients in whom hyperphenylalaninemia is present,^42^ because BH4 deficiency also leads to dysfunction of phenylalanine hydroxylase activity. BH4 is used for the treatment of or as alternative therapy for or in experimental trials of inborn errors in enzymes in BH4 biosynthesis or BH4 recycling such as BH4-responsive phenylketonuria, GTP-cyclohydrolase I deficiency, mild and severe forms of 6-pyruvoyl-tetrahydropterin sysnthase deficiency and pterin-4a-carbinolamine dehydratase deficiency.^11^ Since BH4 is an essential cofactor in the production of the neurotransmitters, 5-HT and dopamine, and also in the generation of NO by NO-synthase, a vast range of vascular and neural disease states have been recognized to potentially benefit from effective BH4 supplementation. 43 However, BH4 does not easily cross the blood brain barrier and cannot be used to correct the central neurotransmitter deficiency in any of the BH4 deficiencies.^42^ In these patients correction of neurotransmitter deficiency is achieved by administering L-dopa and 5-HTP,^42^ which bypass the metabolic block and are converted to dopamine and 5-HT, respectively. These precursors are normally given together with peripheral AADC inhibitor that prevents peripheral decarboxylation of the pre-cursors and allows the precursors to enter the brain.^27^ 5-HTP is used for the treatment of or as alternative therapy for or in experimental trials of disorders such as dihydropteridine reductase deficiency and sepiapterin reductase deficiency.^11^

The consequence of BH4 supplementation provided a hypothesis that administered BH4 might have extremely low efficiency of uptake and/or short retention period. BH4 might not enter cells merely by passive diffusion owing to its hydrophilic nature. Indeed, BH4 is much less permeable across the cell membrane than sepiapterin or dihydrobiopterin, the precursors of the BH4-salvage pathway. It is likely that special mechanisms maintain a steady endogenous level of BH4 in the cells, and exploration of such mechanisms serves for efficient biosynthesis of 5-HTP. Therefore, some mechanisms for BH4 transport were studied.

**Figure 1.**
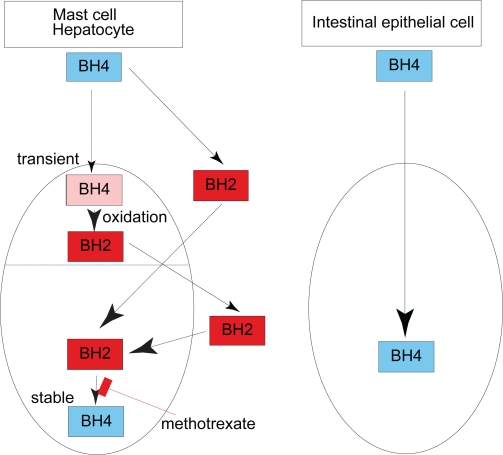
Cell-type specific biopterin transport into the cell. Intestinal epithelial cells (Caco-2) takes up BH4 in its reduced form and releases it from the other side of the polar cell body. In contrast, 5-HT-producing mast cell (RBL2H3 cells) and phenylalanine-metabolizing hepatocyte (primary culture) rapidly take up BH4. Then, BH4 is immediately oxidized to BH2 and is exported back to the extracellular space. Finally, BH2 in the extracellular space enters the cells and contributes to the production of BH4.

Using RBL2H3 cells, a 5-HT-producing mast cell line, BH4 which was transiently taken up appeared to be oxidized to dihydrobiopterin (BH2) and then released. Cells virtually do not take up BH4 in its reduced form, but they do take up BH2 produced from the administered BH4 and convert it back to BH4 through the salvage pathway; a process which was shown to result in an apparent accumulation of BH4. Consequently, BH4 accumulation in RBL2H3 cells was characterised by its sensitivity to methotrexate, an inhibitor of dihydrofolate reductase.^44^,^45^ This machinery was also the case in the hepatocytes because accumulation of BH4 in hepatocytes was almost completely inhibited by methotrexate.^46^ The outline of this feature of BH4 accumulation is depicted in Figure 1.

This model was also supported *in vivo.*^47^,^48^ When mice were treated with 6*R*-BH4, BH2 or sepiapterin either via the oral or intraperitoneal route, it was found that sepiapterin was able to increase tissue BH4 levels most efficiently; a smaller increase in tissue biopterin levels after oral administration of equivalent doses of 6*R*-BH4 and BH2 was also observed. The dihydrobiopterin surge seen after BH4 treatment suggested that systemic oxidation of the administered BH4 occurred before accumulation of BH4 in the tissues. This idea was supported by the following observations: 1) Increase in tissue BH4 was effectively inhibited by methotrexate. 2) When the unnatural diastereomer 6SBH4 was administered to mice, a large proportion of the recovered BH4 was in the form of the 6R-diastereomer, suggesting that this BH4 was the product of a dihydrofolate reductase process by which 7,8-dihydrobiopterin, a non-chiral BH2, converts to 6RBH4. The exogenous BH4 is oxidized and the resultant BH2 circulates through the tissues, and then it was incorporated by various other tissues and organs through a pathway shared by the exogenous sepiapterin and BH2 in their uptake. Thus, maintaining endogenous BH4 in tissues under ordinary conditions is largely dependent on a methotrexate-sensitive process. Together, in addition to intracellular BH4 produced by *de novo* synthesis, BH4 can be also generated from sepiapretin in the extracellular space by salvage pathway and from BH2 taken up from the extracellular space (Fig. 1). BH2,which is present in the extracellular space enters the cells. Exogenous sepiapterin, if available, is also able to enter the cells. Both sepiapterin and BH2 taken up from the extracellular space contribute to the production of BH4 in the cells.^44^,^45^

On the other hand, recent studies on intestinal absorption of BH4 demonstrated that in mice, orally administered BH4 was absorbed in the small intestine as efficiently as BH4 injected in the peritoneal cavity.^47^,^49^ To support this finding, rapid trans-cellular transport of BH4 was observed across monolayer culture of Caco-2 cells of intestinal epithelial origin on filter membrane.^50^ Furthermore, 6SBH4, a synthetic diastereomer of BH4, was loaded into Caco-2 cells and the accumulated BH4 was identified as 6SBH4.^46^ These results provided strong evidence that BH4 had directly accumulated in Caco-2 cells. The BH4 in the cells is immediately released from the cells, and hence the process is insensitive to methotrexate. Thus, the mechanism of BH4 transport across plasma membrane of Caco-2 cells is distinctive from that of RBL2H3 cells. The discovery of distinct mechanisms of BH4 transport among various cells in peripheral organs might have a potential to open up new approaches that regulate the amount of 5-HTP in the gut.

## Specific Role of 5-HTP

It has been generally assumed that 5-HTP, a 5-HT precursor, is immediately decarboxylated to 5-HT and consequently little 5-HTP exists.^20^ However, a report presented an interesting finding on 5-HTP in an immune cell. 5-HT protected NK cells from monocyte-mediated apoptosis and suppression of cytotoxicity and maintained the activation of NK cells induced by interleukin-2, and these protective effects revealed that 5-HT scavenged reactive oxygen species derived from the myeloperoxidase system. Interestingly, 5-HTP shared the scavenger activity with 5-HT; however, the potency of 5-HT was greater by more than 10-fold when compared to 5-HTP in protecting NK cells against functional inhibition and apoptosis.^21^ This observation raises a possibility that exogenous 5-HTP itself as well as the endogenous 5-HTP might function in the extra-neuronal cells. This hypothesis was tested using the macrophages. Evidence suggests that 5-HT modulates immune functions through several 5-HT receptors including 5-HT_1A_ receptor.^51^ For instance, the application of 5-HT induced an increase in macrophage phagocytosis that is blocked by the 5-HT_1A_ receptor antagonist WAY100635,^52^ indicating that exogenous 5-HT contributes to phagocytosis and the enhancing effect is partly mediated by the 5-HT_1A_ receptor. Peritoneal macrophages collected from mice after stimulation with thioglycollate, an activator of macrophages, were found to express TPH protein.^22^ Consistently, these cells had 5-HTP endogenously. Nevertheless, either exogenous or endogenous 5-HTP did not enhance the phagocytic activity *in vitro.*^22^

Although the role of 5-HTP in the phagocytic activity of macrophages was not found, a 5-HT-independent role of 5-HTP was further investigated in the intestinal epithelial cells.^22^ 5-HT has several physiological and pathological implications in the gut as well as in the brain. For example, 5-HT has been reported to increase the rate at which enterocyte precursors proliferate, and the enhancement of enterocyte proliferation by 5-HT might be mediated by a 5-HT2 receptor.^53^ Patients with celiac disease have increased 5-HT-containing enterochromaffin cell numbers and significantly higher peak plasma 5-HT and platelet 5-HT stores than controls, which correlate with postprandial dyspepsia.^54^ On the other hand, the biological activities of 5-HTP itself have not been essentially explored in the gut. Enormous amounts of 5-HT are produced in the enterochromaffin cells and mast cells.^7^ The expression of TPH was, therefore, expected to be confined to enterochromaffin cells and mast cells in the intestine. Unexpectedly, TPH-1 was also found in the epithelial cells of mucosa in the rodent intestine.^22^ Similar results were obtained in the human duodenum using anti-TPH antibody.^55^ The distribution of TPH-1 inside the cells was not uniform.^22^ TPH-1 was abundant on the apical side of the villi epithelium as well as in other cytoplasmic regions.^22^ Moreover, TPH-1 was found in the brush border in the gut epithelial cells by immunohistochemistry with the isolated brush border fraction.^22^ The distribution of endogenous 5-HTP overlapped with that of TPH-1 on the apical side of the villi epithelium.^22^ Although immunoreactivity for 5-HT was not obtained in the intestinal epithelial cells expressing TPH-1 except for enterochromaffin cells, HPLC analysis showed that Caco-2 of intestinal epithelial origin synthesized small but definite amount of 5-HT *de novo.*^22^

**Figure 2.**
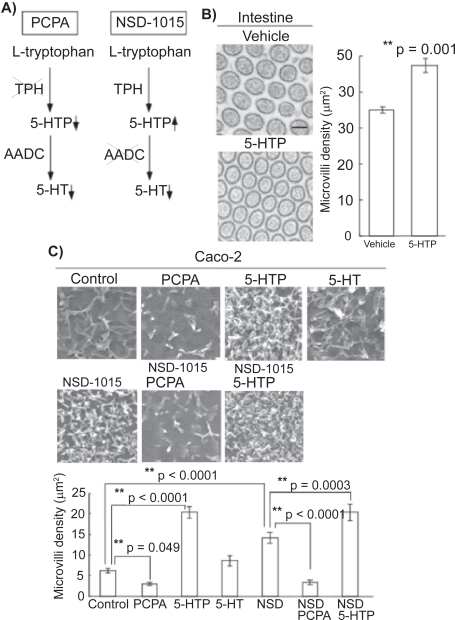
5-HTP regulates the formation of microvilli in the intestine. **A**) Schematic representation of the actions of serotonergic inhibitors. **B**) Transmission electron microscopic analysis of the microvilli of intestine in mice treated with 5-HTP. Scale bar, 100 nm. **C**) Scanning electron microscopic analysis of Caco-2 cells treated with 5-HTP, 5-HT, PCPA, 5-HTP plus PCPA, NSD-1015, NSD-1015 plus 5-HTP, and NSD-1015 plus PCPA. The density of microvilli was quantitatively estimated. Reprinted from *Am J Pathol.* 2008;172:333–344 with permission from the American Society for Investigative Pathology.

Intriguingly, the functions of endogenous 5-HTP as well as exogenously-applied 5-HTP was shown using inhibitors of 5-HT synthesis cascade (Fig. 2A). Analyses with electron microscopy revealed that systemic application of 5-HTP increased the density of microvilli in the mouse intestine *in vivo* (Fig. 2B). That was also the case in the microvilli of Caco-2 cells (Fig. 2C). Exogenous 5-HTP, which can enter the cells, increased the density of microvilli; however, exogenous 5-HT did not. In addition, when exogenous 5-HTP was applied together with p-chlorophenylalanine (PCPA), a TPH inhibitor, the density was also increased, indicating that exogenous 5-HTP plays a role without the effect of endogenous 5-HTP. To confirm the specific role of exogenous 5-HTP in the development of microvilli without the effect of 5-HT, 3-hydroxybenzylhydrazine (NSD-1015), an AADC inhibitor, was used (Fig. 2A). NSD-1015 blocks the synthesis of 5-HT and induces the accumulation of 5-HTP (Fig. 1A). When 5-HTP was given together with NSD-1015, the density was higher than when NSD-1015 was given alone. Thus, the effect is dependent on exogenous 5-HTP. The roles of endogenous 5-HTP were then assessed. Application of PCPA resulted in a decrease in the density of microvilli. To distinguish 5-HTP from 5-HT, NSD-1015 was used. An increase in the density was observed after the application of NSD-1015. Furthermore, the addition of PCPA together with NSD-1015 resulted in a lower density than the addition of NSD-1015 alone. These results suggest that endogenous 5-HTP also plays a role in the density of microvilli. It is still unknown whether the small amount of 5-HT produced in the gut epithelial cells might contribute to the functions of the gut such as gastrointestinal motility and absorption via 5-HT receptors.

## Conclusion

Physiological roles of 5-HTP in the brain have not been reported. On the other hand, 5-HTP has a specific function in the gut. As a unique BH4 transport mechanism, BH4 that transiently enters cells can be rapidly oxidized to BH2 and is exported back to the extracellular space. Meanwhile, the intestinal epithelial cells take up BH4 as its reduced form. Therefore, the intestine shares a unique BH4 transporter mechanism and a specific function of 5-HTP. Further studies would clarify the intestine-specific machinery linking the specific mechanism of BH4-dependent 5-HTP production to the specific function of 5-HTP. A 5-HT precursor 5-HTP is sometimes administered to patients with metabolic disorder.^27^ The finding on the function of 5-HTP in the intestine might create an opportunity to explore the effects of exogenously-applied 5-HTP on the intestine in man.
